# An experimental and numerical study of twin dowel type shear connector

**DOI:** 10.1038/s41598-023-30005-3

**Published:** 2023-02-21

**Authors:** Patricia Vanova, Daniel Dubecky, Michala Weissova, Jakub Bartus, Vincent Kvocak

**Affiliations:** 1 Center of Research and Innovation in Construction, Institute of Structural Engineering, Civil Engineering Faculty, Technical University of Košice, 042 00 Košice, Slovakia; 2 Department of Load Bearing Structures and Intelligent Adaptive Structures, Institute of Structural Engineering, Civil Engineering Faculty, Technical University of Košice, 042 00 Košice, Slovakia; 3 Department of Structures and Bridges, Faculty of Civil Engineering, University of Žilina, 010 26 Žilina, Slovakia

**Keywords:** Civil engineering, Software

## Abstract

The hunger for a better, more effective, more economic, easier for construction, and overall more sustainable bridge design is enormous nowadays. One of the solutions for the problems described is a steel-concrete composite structure with embedded continuous shear connectors. Such structure uses advantages of both materials (concrete in compression and steel in tension) while lowering the overall height of the structure and time of the construction. This paper presents a new design of the connector of twin dowel type, with clothoid dowel, where two dowel connectors are welded longitudinally by flanges to create one twin connector. Its geometrical attributes are closely described and the design origin is explained. The study of the proposed shear connector consists of experimental and numerical part. In experimental study, the experiments performed—four push-out tests—and their setup, instrumentation and material characteristics are described and their results in a form of load-slip curves are presented and analyzed. In numerical study the finite element model developed in ABAQUS software is presented with a detailed description of the modeling process. In results and discussions the results of the numerical study are compared with results from the experimental study and the resistance of the proposed shear connector is briefly compared to shear connectors from chosen studies.

## Introduction

**Figure 1:**
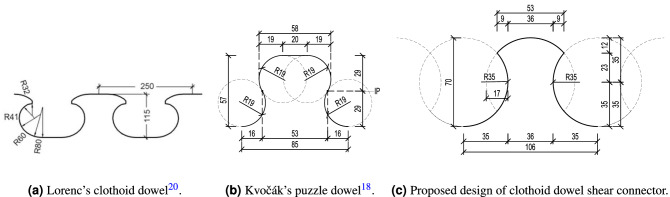
The development of the design.

The continuous shear connector is a widely studied type of the shear connection between the researchers of composite bridges. Lorenc et al. did a thorough study of the dowel shape, concluding with a clothoid design (see Fig. 1a)^1^. They determined the failure mode of the clothoid based on the push-out tests and numerical study using ABAQUS software^2^. Berthellemy et al. did a short comparison of puzzloid, clothoid and shark dowel shape, in close cooperation with Lorenc et al.^1,2^, which resulted preferably for the clothoid due to the higher shear resistance and easier fabrication. This was caused by lower stress at the dowel basin^3–5^. Lechner et al. studied behavior of Ultra-High Performance Concrete (UHPC) with embedded dowels in concrete beams of different thickness. The dowels used were clothoid type, adapted from Lorenc’s research. Their study showed the very high load carrying capacity up to the thickness of 40 mm^6^. Classen and Hegger focused on pry-out failure of two dowel types—clothoid and puzzloid^7–9^. Based on their research they opposed an approved shear resistance equation in Germany with their solution^8^.

Huang et al. studied the puzzloid dowels with elliptical holes and therefore combining the advantages of both dowel and perfobond shear connector. The holes increased the shear resistance of the proposed connector significantly. The failure mode was caused by concrete shear failure and no failure of steel dowels occurred^10^.

Twin connectors were typically studied on perfobond type shear connection. Ahn et al. studied twin perfobond connectors with 55 mm holes. They compared twin ribs with three different distances in between them and analyzed the results, focusing on crack development. With regression analysis they proposed an adjusted equation better fitted for twin rib perfobond shear connectors^11,12^. Deng et al. compared the twin perfobond connectors (T-PBL) to the channel and angle shear connectors, with channel connector concluding with the highest shear resistance^13^. Changyu et al. did an experimental study concentrated on transverse flexural resistance of the T-PBL connectors. Their study presented different failure modes based on the type of perforated rebars^14^. Cândido-Martins invastigated two side by side perfobond connectors with comparison to single connector. Their results showed increase in bearing capacity, however not to a point of twice the value of the single perfobond connector^15^. Hai et al. studied the effect of the parallel placing of the single perfobond connectors and revised a formula used for the single connectors to take into account such placing^16^.

The goal of this research was to propose a new shape of a clothoid dowel shear connector (Fig. 1c), study its behavior by experimental and numerical study, determine its shear resistance, and prove suitability of the twin dowel type connector as an alternative to both the single dowel shear connectors as well as twin perfobond shear connectors.

## Experimental study

### Shear connector design

The dowel shape was created based on previous research^1,17–20^ and it considers advantages of those research. Lorenc’s research^1^ suggested the clothoid (see Fig. 1a) as a better solution in practice in comparison with puzzle shaped connector due to the problems with shear connectors separation in some types of production^17^. Kvočák’s research proposed a puzzle-type dowel with an inflection point (IP, see Fig. 1b) in between two equal circles with higher teeth to base ratio as well as higher dowel height to dowel distance ratio^18^.

**Figure 2:**
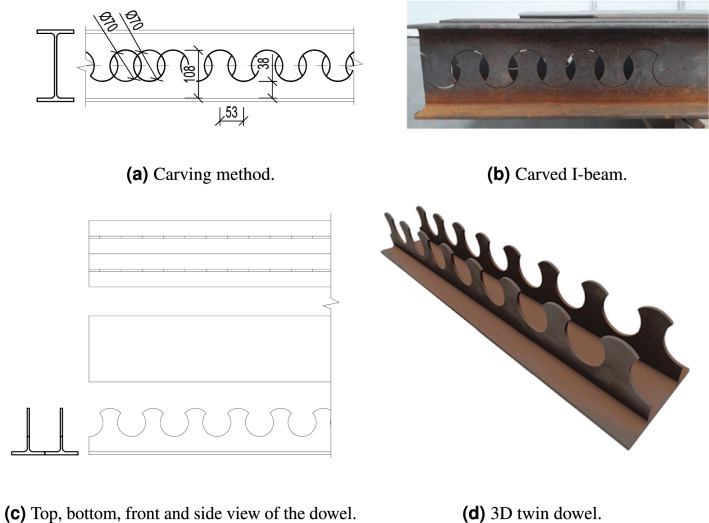
Design of the twin dowel.

This paper presents a clothoid-type twin dowel connector with a greater radius to the connector height ratio. The dowel is created by two overlapping circles with approximately 24% overlap (see Fig. 1c), where the overlapping parts are removed from its geometry to create the clothoid dowel shape. Its aim is to enlarge the diameter of the concrete stud formed in between the dowel teeth—in comparison with both Lorenc’s^1^ and Kvočák’s^18^ dowel proposals (see Fig. 1), which should increase the shear resistance of the same height dowel. Further, the larger diameter causes a distribution of the stress over a larger edge area and thus reduces the stresses at the connector edges, which should cause later crack initiation under a fatigue loading^19,20^.

The connector geometry is created by longitudinally cutting an I-beam which creates two T-beams with the dowels on top. (see Fig. 2a,b). Both sides of the beam are then used to form the twin type connector by welding the flanges longitudinally (see Fig. 2c,d).

### Test setup

**Figure 3:**
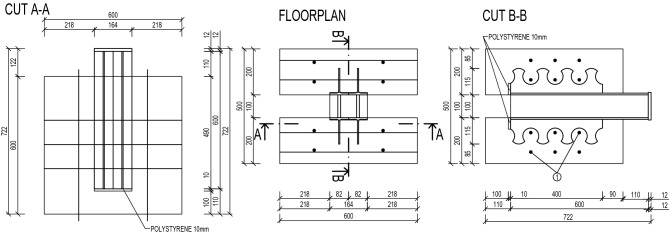
Layout of the twin dowel type shear connector for push-out tests.

Experimental study consisted of four push-out tests (marked PT-S-1 to 4). Principle and course of experimental tests was identical to the previous experiments performed in the Center of Research and Innovation in Construction^21^ and followed general recommendations of Eurocode 4^22^ for specific push tests. The shear connectors carved from IPE 160 (see Fig. 2a,b) were welded onto the middle steel structure, which served for load transfer, and then embedded in two concrete blocks of 600 $\times$ 600 $\times$ 100 mm dimensions, placed on 10 mm polystyrene in order to prevent the steel pushing directly into concrete. Transverse reinforcement was placed in between the individual dowels with a minimum of 10 mm coverage so it would not interfere with the steel dowels after the 10 mm slip. The layout and exact measurements are visible in Fig. 3.

**Figure 4:**
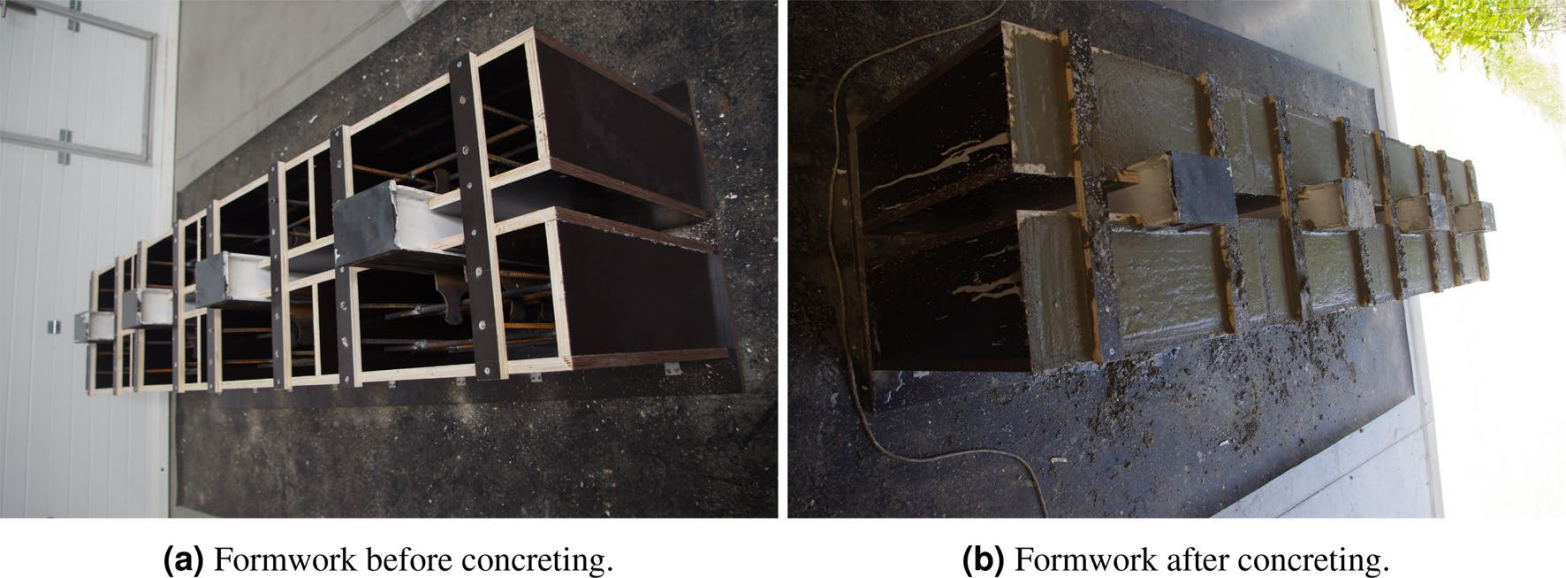
Specimens preparations for the push-out tests.

The formwork of the specimens was put on a rubber mat to prevent damages to the floor. The concrete slabs were concreted in an upright position (see Fig. 4) due to the technological reasons—for both slabs to be concreted at the same time. The upright position during concreting of continuous shear connectors was previously also used by Vianna et al.^23^ and Deng et al.^13^ as an uneven grain along the height of the concrete slab does not significantly affect the shear resistance of the continuous shear connectors.

**Figure 5:**
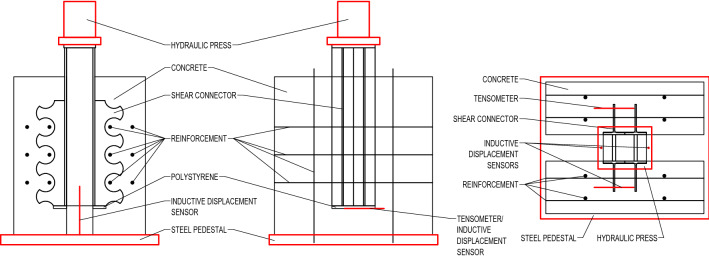
Push-out test setup and instrumentation.

Specimens prepared in this way were subsequently, after approximately four month of currying, put into a hydraulic press (actuator INSTRON) on a cleaned steel pedestal in a centered position (see Fig. 5). The load was applied onto a top sheet plate in several loading conditions (LCs)—initially from 0 to 10 to 20 kN, then in 20 kN increments up to 380 kN, after which a loading cycle of 50 LCs was performed, going down and up from 380 to 50 kN. After the last cycle, the load was increased to 400 kN and then it was increased by 50 kN increments until the specimens reached its plasticity, with exception of first two specimens (PT-S-1 and PT-S-2), where the load above 800 kN was increased in 10 and 20 kN increments (visible in Fig. 8) in order to better determine the breaking point. After the slip in the specimens surpassed the 10 mm point (equal to thickness of the polystyrene, see Figs. 3 and 5), the load increasement was stopped and the specimens were unloaded in three LCs—in approximately 1/2 and 1/10 of the maximum load, respectively, and zero.

**Figure 6:**
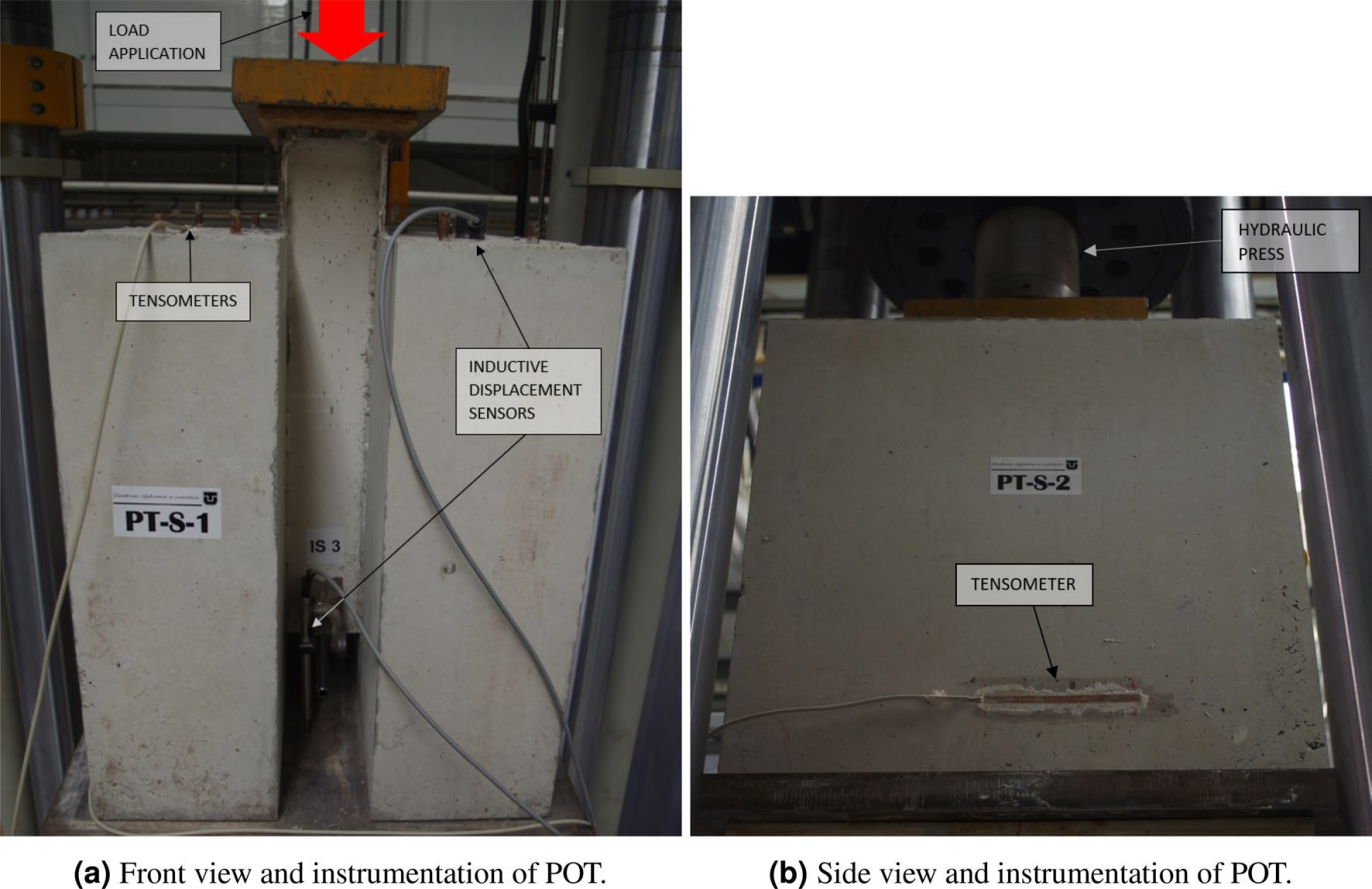
Experimental study.

The loading force was precisely measured and recorded by the software that controlled the hydraulic press (WaveMatrix). The slip was measured by two inductive displacement sensors placed in between the concrete blocks near the middle steel structure from both sides (see Figs. 5 and 6). Additionally, the crack initiation of concrete blocks was measured on first two specimens by tensometers and inductive displacement sensors placed at the bottom of the outer side as well as on the top of the concrete blocks, moved from center axis to the side so that the center of the measuring device would be placed directly above peaks of one of the dowels (see Figs. 5 and 6).

### Material properties

**Figure 7:**
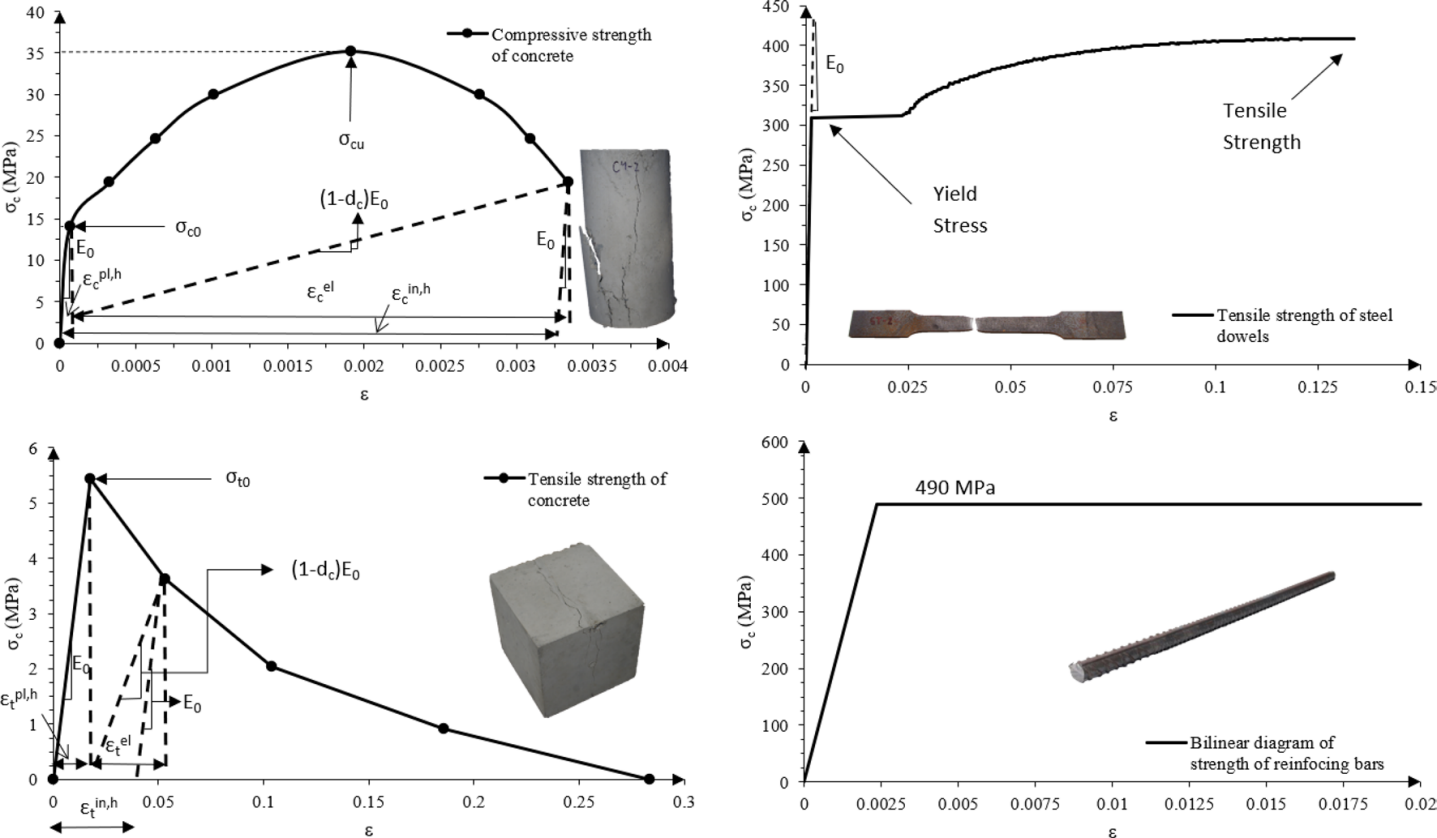
Material properties of steel and concrete.

The specimens were made out of two main materials—concrete and steel. Concrete C30/37 and steel S275 of European quality were used. In order to precisely determine the material properties for analytical and numerical study, they were tested—compressive, tensile and flexural tests of concrete, and tensile tests of steel were performed in accordance with Eurocode standards^24–29^. At the time of the experimental study, the compressive and tensile strength of concrete were 35.2 and 5.4 MPa, respectively. The Yield Point of steel was 309.5 MPa. The results of the material testing are shown in Fig. 7.

Additionally, the concrete blocks were also reinforced with reinforcement marked B500B with 12 mm diameter. The non-rigid reinforcement was not tested and therefore the normative values^30^ were used in further studies.

### Test results and discussion

**Figure 8:**
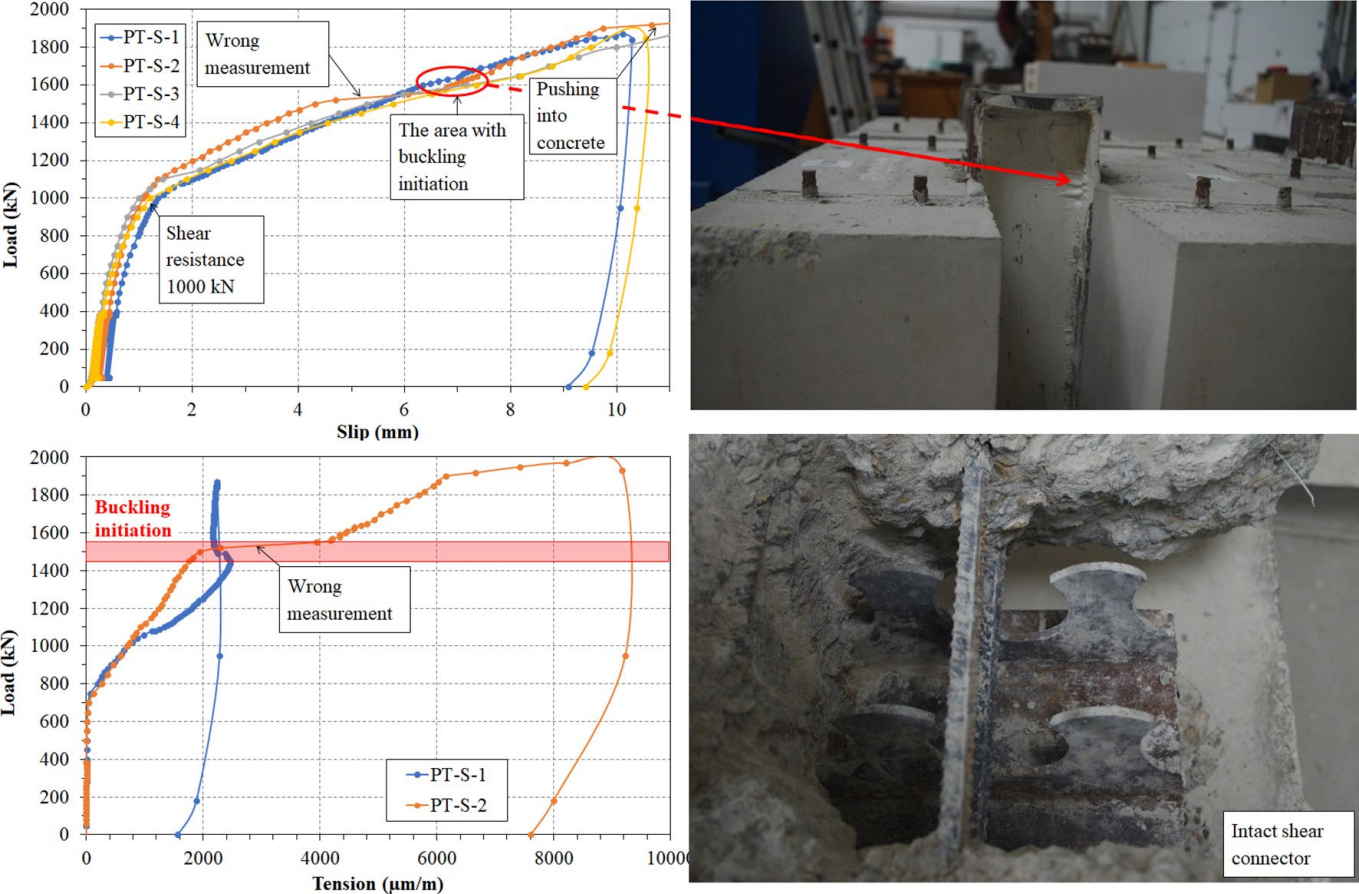
Results of the push-out tests.

Behavior of the specimens was as expected during the initial phase of its elastic behavior–linear with a minor slip gain (1–1.2 mm, see Fig. 8 and Table 1), at the end of which the first cracks started to appear. However during the second, elastic-plastic phase, the unexpected results were measured. After the point of shear resistance, instead of transition into horizontal line in the graph with larger growths of slip, the graph continued with diagonal line with incremental slip increasement (see Fig. 8). At the point of 6 mm the specimens started to develop buckling of the pushed steel part at the edge of the concrete blocks.

The shear resistance was determined at the loading cycle equal to value of 1000 kN (see Table 1). The average maximum load was 1860 kN at the point of aproximattely 10 mm slip.

Fortunately, in two specimens equipped with tensometers the measuring devices were crossed by cracks. From the Fig. 8 it is visible, that the cracks started developing at the point of loading of 750 kN. From that point to the point of buckling, the tension in cracks continuously linearly grew. After buckling started to occur, crack development in one of the specimens stopped, as the forces did not transfer to the lower part of the specimen. However, in second specimen, the tension continued to grow, which was also shown by the increase in crack width (see Fig. 8).

**Table 1:** Test results.

Name	*P*$_{Rk}$ (shear resistance)	Slip at *P*$_{Rk}$	*P*$_{max}$ (maximum load)	Slip at *P*$_{max}$
PT-S-1	1050 kN	1.57 mm	1870 kN	10.1 mm
PT-S-2	1000 kN	0.99 mm	1970 (1920) kN	13.8 (10.7) mm
PT-S-3	1000 kN	0.99 mm	1850 (1800) kN	12.6 (10.0) mm
PT-S-4	1000 kN	1.21 mm	1850 kN	10.6 mm

The experimental study was preceded by an analytical study. The theoretical elastic load-bearing capacity of the tested specimen was counted from an Eq. (1) proposed by Rovňák et al.^31^, and based on Bijlaard et al.^32^ and Kraus et al.^33,34^, with the result of 478.565 kN.

1 $$\begin{aligned} P_{u, t e o r}=11.83 h_{k} t f_{c m} \end{aligned}$$

where *P*$_{u, t e o r}$ is the shear resistance, *h*$_{k}$ is the height of the shear connector, *t* is the thickness of the shear connector and *f*$_{c m}$ is the mean value of cylindrical compressive strength of concrete.

As mentioned above, the experimentally measured shear resistance more than twice surpassed the resistance from analytical calculations. For this reason, the load incrementation in the experiments of the initial specimens is lower than of the later ones.

After the experiments, the specimens were disassembled in order to determine the failure part. No visible deformation were developed by steel dowels, steel flanges or reinforcement, and therefore it is safe to assume the shear resistance surpasses the measured values with greater strength of concrete. Concrete C30/37 was chosen as based on the analytical study such great resistance was not expected, as well as due to its usage for shrot-span composite bridges. Concrete blocks developed several cracks on the outer sides (Fig. 10), which were the reason of the specimens failure. The particular concrete failure mode explains the unexpected behavior observed during the testing. Since the sole failure were the cracks in the concrete slabs, slip grew linearly as they widened.

## Numerical study

The 3D specimen was created in the ABAQUS software—a software based on finite element method, with only quarter of the real specimen modeled, using the symmetry of the specimen to its advantage. Similar practice is very common in finite element analysis as it shortens the computation time (see Lorenc^1^, Kim et al.^35^ and Bezzera et al.^36^). The model did not include any imperfections in its geometry.

### Material properties and behavior

Material properties of steel and reinforcement were specified in the numerical study by three material options provided by ABAQUS software—Density, Elastic behavior and Plastic behavior. They are shown in the Fig. 7 and Table 3. In order to specify both compressive as well as tensile material behavior of conrete, the suboption ’Concrete damaged plasticity’ (CDP) was chosen. As only bilinear diagrams of concrete strength were obtained from material testing, the compressive and tensile diagrams were created based on Allam et al.^37^ and Hafezolghorani et al.^38^, where the uniaxial compressive behavior was specfied by the Eqs. (2), (3) and (4), and the uniaxial tension behavior by Eqs. (5), (6) and (7). The material properties of concrete used in numerical study are visible in Fig. 7 and Table 2.

For all the material characteristics necessary for numerical study which were not obtained experimentally (Poisson’s Ratio of both steel and concrete and density and Young’s Modulus of steel), the normative values obtained from Eurocode 2 and 3^29,30^ were used.

Uniaxial compressive behavior

2 $$\begin{aligned}{} & {} \sigma _{c}=\left( 1-d_{c}\right) E_{0}\left( \varepsilon _{c}-\varepsilon _{c}^{p l, h}\right) \end{aligned}$$

3 $$\begin{aligned}{} & {} \left\{ \begin{array}{l} \varepsilon _{c}^{i n, h}=\varepsilon _{c}-\frac{\sigma _{c}}{E_{0}} \\ \varepsilon _{c}^{p l, h}=\varepsilon _{c}-\frac{\sigma _{c}}{E_{0}}\left( \frac{1}{1-d_{c}}\right) \\ \end{array}\right. \end{aligned}$$

4 $$\begin{aligned}{} & {} \varepsilon _{c}^{p l, h}=\varepsilon _{c}^{i n, h}-\frac{d_{c}}{\left( 1-d_{c}\right) } \frac{\sigma _{c}}{E_{0}} \end{aligned}$$

where $\sigma _{c}$ is uniaxial compression stress, $d_{c}$ is scalar damage variable in compression, $E_{0}$ is Young’s Modulus, $\varepsilon _{c}$ is strain compression, $\varepsilon _{c}^{p l, h}$ is plastic hardening strain in compression, $\varepsilon _{c}^{i n, h}$ is inelastic compression strain^37,38^.

Uniaxial tensile behavior

5 $$\begin{aligned}{} & {} \sigma _{t}=\left( 1-d_{t}\right) E_{0}\left( \varepsilon _{t}-\varepsilon _{t}^{p l, h}\right) \end{aligned}$$

6 $$\begin{aligned}{} & {} \left\{ \begin{array}{l} \varepsilon _{t}^{c k, h}=\varepsilon _{t}-\frac{\sigma _{t}}{E_{0}} \\ \varepsilon _{t}^{p l, h}=\varepsilon _{t}-\frac{\sigma _{t}}{E_{0}}\left( \frac{1}{1-d_{t}}\right) \\ \end{array}\right. \end{aligned}$$

7 $$\begin{aligned}{} & {} \varepsilon _{t}^{p l, h}=\varepsilon _{t}^{c k, h}-\frac{d_{t}}{\left( 1-d_{t}\right) } \frac{\sigma _{t}}{E_{0}} \end{aligned}$$

where $\sigma _{c}$ is uniaxial tension stress, $d_{t}$ is scalar damage variable in tension, $E_{0}$ is Young’s Modulus, $\varepsilon _{t}^{p l, h}$ is plastic hardening strain in tension, $\varepsilon _{t}^{ck, h}$ is cracking strain^37,38^.

**Table 2:** Behavior of concrete in finite element analysis.

Material behavior	Value	Unit
Density	2.27E−009	tonne/mm$^{3}$
Elastic		
Young’s modulus	30 646	MPa
Poisson’s ratio	0.2	–
Concrete Damaged plasticity		
Compressive strength	35.232	MPa
Tensile strength	5.428	MPa

**Table 3:** Behavior of steel in finite element analysis.

Material behavior	Value	Unit
Density	7.85E−009	tonne/mm$^{3}$
Elastic		
Young’s modulus	210,000	MPa
Poisson’s ratio	0.3	–
Plastic		
Yield point	309.502	MPa
Tensile strength	408.653	MPa

### Interaction, loading and boundaries, step and mesh

**Figure 9:**
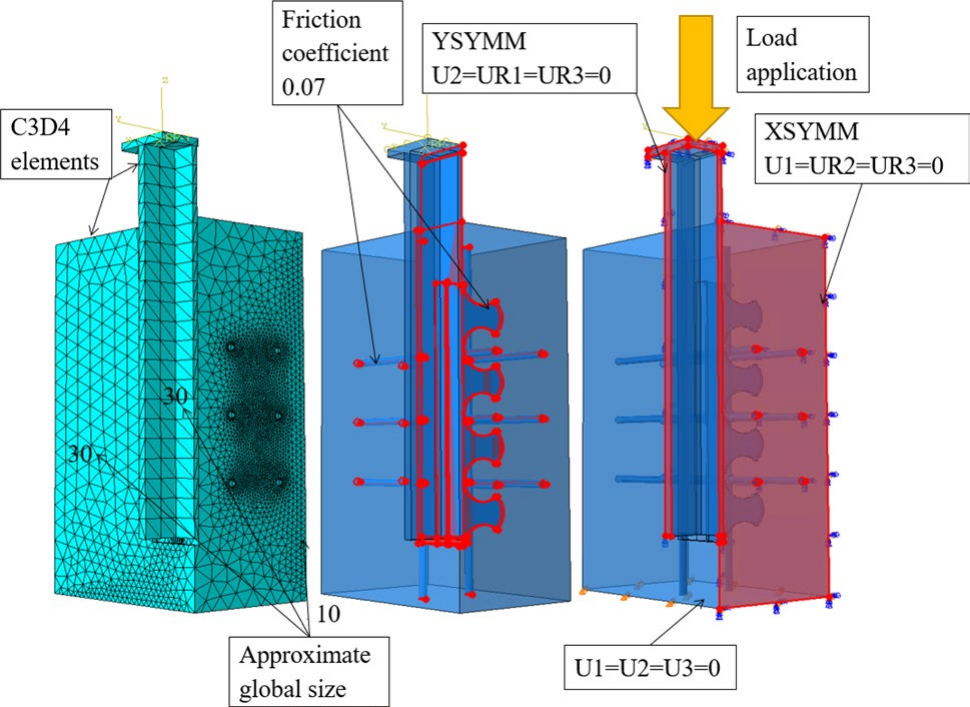
ABAQUS software specifications of mesh, interaction and loading and boundary conditions settings.

Interaction in between all interacting parts (steel strip and concrete, and reinforcing bars and concrete) was defined in the numerical model via General contact option with ’all with self’ surface pairs preventing the parts to penetrate each other. Additionally, in between those parts, the individual property assignments were set, specifying the friction coefficient to a value of 0.07 (Fig. 9).

In ABAQUS’s load module, the boundaries simulating the steel pad of hydraulic press as well as surfaces of symmetry were set. The bottom of the concrete part was pinned—prohibiting movement in any direction (Fig. 9). The surfaces of symmetry were defined with XSYMM, YSYMM boundary conditions for X and Y axis of symmetry, respectively.

Loading in the numerical model copied the experimental loading up to the point of bucking. An interval of 104 loading cycles was put into software via an amplitude with maximum value of 1 being equal the buckling point of 1500 kN. An amplitude time was equal to step time. The load was add in as a concentrated force with a reference point in the middle of the upper steel sheet plate, which had a constraint ’rigid body’ that secured the non-deformability of the defined area and therefore allowed the force transfer into the specimen.

The finite element analysis was performed using a Dynamic, Explicit step. This step includes in its finite element method equation acceleration and velocity phenomenon (see Eq. 8), which are a source of inertia force in the simulation, which can cause disturbances in the results^39^ as it is an unwanted phenomenon. Nevertheless, if set up with low speed of loading, the dynamic step can be used for static simulations as it also was in many previous research and numerical studies of push-out tests, such as in Bezzera et al.^36^, Lima et al.^40,41^ and Nguyen and Kim^42^, and is generally recommended for problems requiring specific contact conditions^39^. In the step setting, the non-linear geometry option was chosen to allow an occurrence of non-linearities in the simulation from the specimen deformation. The total step time was 0.1s.

8 $$\begin{aligned}{}[M]\{a\}+[C]\{v\}+[K]\{u\}=\{F\} \end{aligned}$$

where *[M]* is mass matrix, $\lbrace a \rbrace$ is acceleration, which equals to $\lbrace u \rbrace$”, *[C]* is damping matrix, $\lbrace v \rbrace$ is velocity, which equals to $\lbrace u \rbrace$’, *[K]* is stiffness matrix, $\lbrace u \rbrace$ is displacement and $\lbrace F \rbrace$ is force^43^.

Another setting specified in the step module was a mass scaling. The purpose of this setting is to lower the time of the computation. After the implementation of the load at the beginning of this step, the elements bordering the top sheet plate are exposed to large stresses that cause large deformation. By decreasing the lengths of the edges of the deformed elements, and therefore also decreasing the mass of the elements, the necessary time increment is also lower, leading to an overall lower computation time^39^. The mass scaling was applied to the whole model at the beginning of the dynamic, explicit step, with the scale to target time increment of 1E−007.

Meshing was done by tetrahedron elements C3D4. Such elements can have a trouble converging^39^, but since the size of the model was eliminated by three quarters, no problems with the convergence occurred during the process of numerical modeling. The entire model had approximate global size of the elements set to the value of 30 mm, with an exception of the outer concrete surface, where most of the defying cracks of breaking failure occurred, where the approximate global size was lowered to 10 mm. Due to the round shape of the dowels, which could not be simplified, a maximum deviation factor played a big role in creating mesh of the model as well. It’s value was 0.03 throughout the model. This number equals to a distance between element edge and the real edge of the curvature divided by the length of the element edge^39^.

## Results and discussion

**Figure 10:**
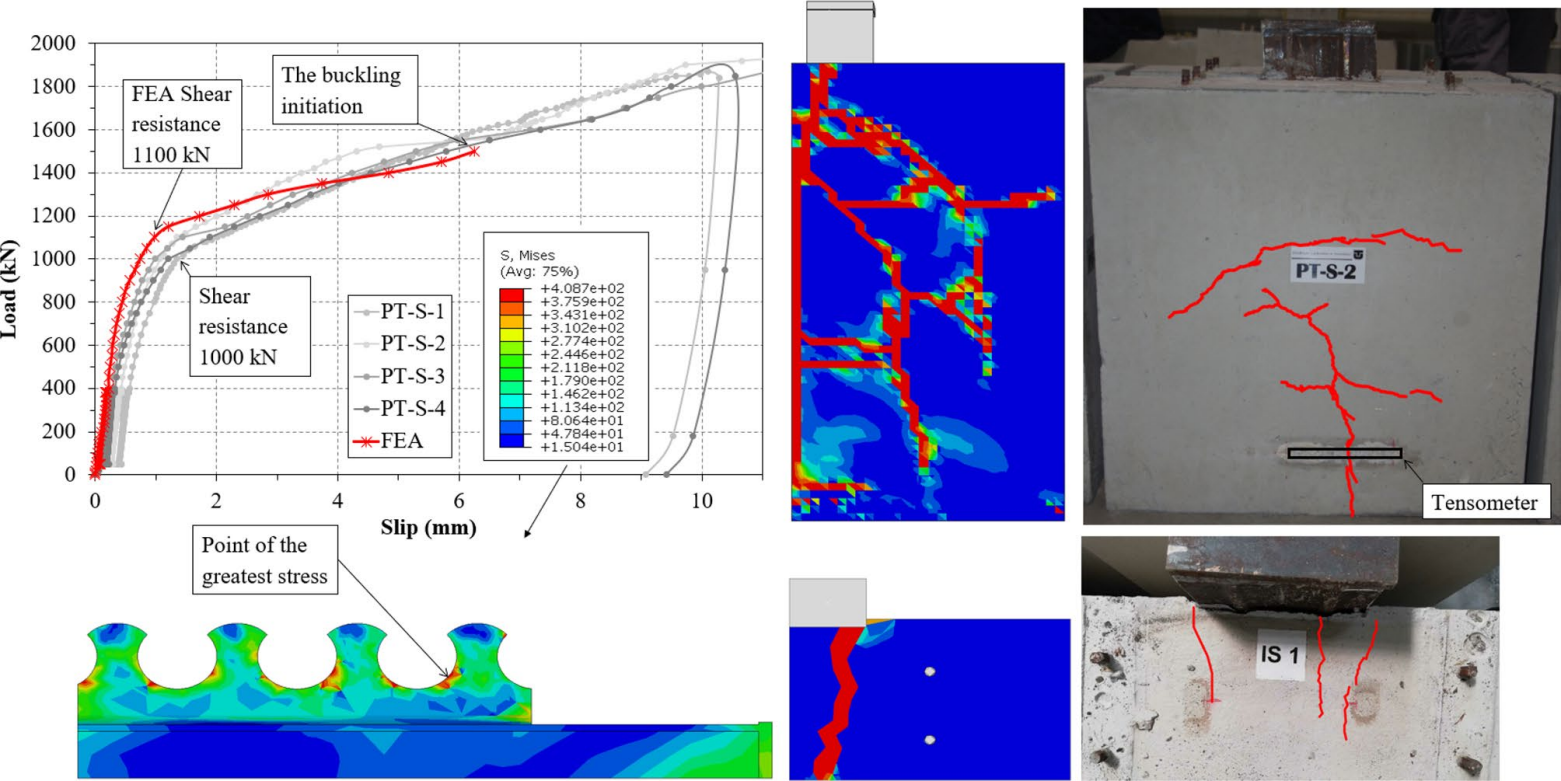
Results of the finite element analysis.

The behavior of the numerical model closely followed the behavior of the specimens during push-out tests. The shear resistance of the FEM model slightly surpassed the shear resistance found by experimental study at the point of 1100 kN (see Fig. 10 and Table 4). The numerical study developed concrete cracks almost identical to the cracks developed in the specimens tested, which generally initiated in parallel with one of the shear strips. No damages or deformations of the steel connector occurred. The failure mode observed in numerical study supported the conclusions from experimental testing—after the point of shear resistance, the slip continued to linearly increase with greater increments due to the developmnet of concrete cracking. Stress distribution in the shear connector showed the point of the greatest stress (see Fig. 10) at the connector basin, and thus the point where the cracks in steel would advance. Based on the internal stresses, tensometers could be carefully placed onto the connector in the future studies.

**Table 4:** Comparison of the analytical/experimental/numerical analysis.

Study	Value (kN)	Ratio (value/experimental)
Analytical	478.565	0.48
Experimental	1000	1
Numerical	1100	1.1

In comparison with other dowel connectors, the proposed twin dowel connector performed well, considering the beams overall low height and thickness (see Table 5). When comparing the twin shear connectors, the perfobond type performs better in longitudinal shear connection. However, the twin dowel connector brings the advantage of single dowel connectors in comparison to perfobond—its simple assembly during construction, and therefore, it is a suitable alternative.

**Table 5:** Comparison with different connectors.

Author	Type	Steel/concrete strength	Connector dimensions in POT (dowel) height/thickness/length	POT results
Lorenc et al.^1,2^	Dowel clothoid	414.3 MPa/63 MPa	215 (50) mm/15 mm/900 mm	2300 kN
Kvočák et al.^18^	Dowel puzzle	315.3 MPa/39.7 MPa	87 (57) mm/5.9 mm/400 mm	650 kN
Ahn et al.^11^	Twin perfobond	352.3 MPa/28.1 MPa	129 mm/6 mm/500 mm	2025 kN
Deng et al.^13^	Twin perfobond	400 MPa/40.3 MPa	160 mm/16 mm/360 mm	2465 kN
Vaňová et al.	Twin dowel clothoid	309.5 MPa/35.2 MPa	115 (70) mm/5 mm/400 mm)	1000 kN

## Conclusion

This paper presented analytical, experimental and numerical study of a twin dowel type shear connector. With the equation (Eq. 1) by Rovňák^31^ the theoretical shear resistance of the proposed shear connector was counted. Further, the experimental study consisting of push-out tests and material tests was conducted and the results were analyzed. The finite element analysis of one quarter of a push-out specimen was created in ABAQUS software. The results of the numerical study proved the failure mode determined by the experimental study (concrete cracking) and showed the stress distribution in the clothoid with the maximum stress point at the basin of the dowel.

The inconsistency between the results of the analytical and experimental study calls for further parametric study using the numerical model presented in this paper. The results show a possible dependence of shear resistance onto the distance of the two connectors with a prediction of a formation of concrete block in between the connectors, and thus the width of such block could be the key for more precise equation for twin dowel shear connectors.

To prove the suitability of the proposed twin connector for use in slabs and bridges, further experimental analysis would be necessary, including fatigue tests, four-point flexural tests and dynamic tests.

In conclusion, the analysis presented in this paper showed an innovative type of connector with a new shape of clothoid dowel bringing advantages from previous research^1,18^. The twin connection showed a significant improvement in the shear resistance, and the possible effect of dowel shear connector placement in both slabs and bridges onto its shear resistance.

## Supplementary Information

Supplementary Information.

## Data Availability

The datasets used and/or analyzed during the current study are included in this article or its supplementary file.

## References

[1] Lorenc W (2016). Non-linear behaviour of steel dowels in shear connections with composite dowels: Design models and approach using finite elements. Steel Constr..

[2] Kożuch, M. & Lorenc, W. The behaviour of clothoid-shaped composite dowels: Experimental and numerical investigations. *J. Constru. Steel Res.* **167**. 10.1016/j.jcsr.2020.105962 (2020).

[3] Berthellemy, J., Hechler, O., Lorenc, W., Seidl, G. & Viefhues, E. Premiers rèsultats du projet de recherche europèen precobeam de connexion par dècoupe d’une tôle. *Revue Construction Mètallique* 3–26 (2009).

[4] Berthellemy J, Lorenc W, Mensinger M, Rauscher S, Seidl G (2011). Zum Tragverhalten von Verbunddübeln -Teil 1: Tragverhalten unter statischer Belastung. Stahlbau.

[5] Berthellemy, J., Zanon, R., Seidl, G. & Lorenc, W. An innovative solution for small span bridges - precobeam. *Toronto Innov. Steel Bridges* (2014).

[6] Lechner T, Gehrlein SF, Fischer O (2016). Structural behaviour of composite dowels in thin UHPC elements. Steel Constr..

[7] Claßen M, Gallwoszus J (2016). Concrete fatigue in composite dowels. Struct. Concr..

[8] Classen M, Hegger J (2017). Shear-slip behaviour and ductility of composite dowel connectors with pry-out failure. Eng. Struct..

[9] Classen M, Hegger J (2017). Assessing the pry-out resistance of open rib shear connectors in cracked concrete - Engineering model with aggregate interlock. Eng. Struct..

[10] Huang P, He J, Kong F, Mei K, Li X (2022). Experimental study on the bearing capacity of PZ shape composite dowel shear connectors with elliptical holes. Sci. Rep..

[11] Ahn JH, Lee CG, Won JH, Kim SH (2010). Shear resistance of the perfobond-rib shear connector depending on concrete strength and rib arrangement. J. Constr. Steel Res..

[12] Kim SH, Lee CG, Ahn JH, Won JH (2011). Experimental study on joint of spliced steel-PSC hybrid girder, Part I: Proposed parallel-perfobond-rib-type joint. Eng. Struct..

[13] Deng W, Xiong Y, Liu D, Zhang J (2019). Static and fatigue behavior of shear connectors for a steel-concrete composite girder. J. Constr. Steel Res..

[14] Changyu W (2022). Research on the transverse flexural performance of T-PBL joints between corrugated steel webs and concrete slabs. Structures.

[15] Cândido-Martins JP, Costa-Neves LF, Vellasco PCS (2010). Experimental evaluation of the structural response of Perfobond shear connectors. Eng. Struct..

[16] Hai, N. M., Akinori, N., Naoki, T. & Masaki, O. Experimental study on shear resistance of perfobond strip with parallel perfobond ribs and longitudinal plural perforations. *11th German Japanese Bridge Symposium* (2017).

[17] Seidl G, Müller J (2011). Massive Verbundbrücken für die Bahn. Beton- und Stahlbetonbau.

[18] Kvočák V, Kožlejová V, Dubecký D, Kanishchev R, Vaňová P (2019). Experimental and software analysis of composite action in steel-concrete composite bridges with continuous shear connectors. Ce/Papers.

[19] Rowiński S, Lorenc W, Kozuch M (2014). Study on fatigue cracks in steel - Concrete shear connectors composite dowels MCL. Key Eng. Mater..

[20] Harnatkiewicz P, Kopczyński A, Kozuch M, Lorenc W, Rowiński S (2011). Research on fatigue cracks in composite dowel shear connection. Eng. Fail. Anal..

[21] Vanova P, Orolin P, Dubecky D (2021). Material characteristics of push-out tests. IOP Conf. Ser. Mater. Sci. Eng..

[22] EN 1994-1. Eurocode 4 - design of composite steel and concrete structures - part 1: General rules and rules for buildings. Tech. Rep. (2009).

[23] Da C, Vianna J, De Andrade SA, Da PC, Costa-Neves LF (2013). Experimental study of Perfobond shear connectors in composite construction. J. Constr. Steel Res..

[24] EN 12390-3. Testing hardened concrete - part 3: Compressive strength of test specimens. Tech. Rep. (2010).

[25] EN 12390-4. Testing hardened concrete - part 4: Compressive strength - specification for testing machines. Tech. Rep. (2001).

[26] EN 12390-5. Testing hardened concrete - part 5: Flexural strength of test specimens. Tech. Rep. (2011).

[27] EN 12390-7. Testing hardened concrete - part 7: Density of hardened concrete. Tech. Rep. (2011).

[28] EN 12390-13. Testing hardened concrete - part 13: Determination of secant modulus of elasticity in compression. Tech. Rep. (2014).

[29] EN 1993-1-1. Eurocode 3: Design of steel structures - part 1-1: General rules and rules for buildings. Tech. Rep. (2005).

[30] EN 1992-1-1. Eurocode 2: Design of concrete structures - part 1-1: General rules and rules for buildings. Tech. Rep. (2004).

[31] Rovňák M, Ďuricová A, Kundrát K, Naď Ľ (2006). Spriahnuté oceľovo-betónové mosty.

[32] Bijlaard, F., Sedlacek, G. & Stark, J. Procedure for the determination of design resistance from tests. Backgroud report to Eurocode 3 ”Common unified rules for Steelstructures”. *IBBC TNO-report* (1988).

[33] Kraus D, Wurzer O (1997). Nonlinear finite-element analysis of concrete dowels. Comput. Struct..

[34] Kraus, D. & Wurzer, O. Bearing Capacity of Concrete Dowels. *Compos. Constr. Convent. Innov.* 133–139 (1997).

[35] Kim, K. S., Han, O., Gombosuren, M. & Kim, S. H. Numerical simulation of Y-type perfobond rib shear connectors using finite element analysis. *Steel Compos. Struct.* **31**, 53–67. 10.12989/scs.2019.31.1.053 (2019).

[36] Bezerra LM, Barbosa WC, Bonilla J, Cavalcante OR (2018). Truss-type shear connector for composite steel-concrete beams. Constr. Build. Mater..

[37] Allam SM, Shoukry MS, Rashad GE, Hassan AS (2013). Evaluation of tension stiffening effect on the crack width calculation of flexural RC members. Alex. Eng. J..

[38] Hafezolghorani M, Hejazi F, Vaghei R, Jaafar MSB, Karimzade K (2017). Simplified damage plasticity model for concrete. Struct. Eng. Int..

[39] Dassault Systèmes. Abaqus 6.13 online documentation (2013).

[40] Lima, J., Bezerra, L. M. & Bonilla, J. Comparative analysis between the shear resistance of the Truss Type shear connector and stud bolt. In *Proceedings of the XLI Ibero-Latin-American Congress on Computational Methods in Engineering, ABMEC* (2020).

[41] Lima JM, Bezerra LM, Bonilla J, Barbosa WC (2022). Study of the behavior and resistance of right-angle truss shear connector for composite steel concrete beams. Eng. Struct..

[42] Nguyen HT, Kim SE (2009). Finite element modeling of push-out tests for large stud shear connectors. J. Constr. Steel Res..

[43] Ivančo, V., Kubín, K. & Kostolný, K. *Metóda konečných prvkov I* (elfa, 1994).

