# Supplementary material for: An experimental and numerical study of twin dowel type shear connector
Source: Sci Rep. 2023 Feb 21;13:3071. doi: 10.1038/s41598-023-30005-3 (PMC9945454; doi:10.1038/s41598-023-30005-3)
Supplement: Supplementary file 1 — Supplementary Information. [file 41598_2023_30005_MOESM1_ESM.zip › Raw_data/Beams.pdf]

SPRIEVODNÝ LIST ZÁKAZKY
č.:

Druh skúšky: Pevnosť v ťahu pri ohybe
Pevnosť v priečnom ťahu vzoriek tvaru hranol

Skúšobný postup č.: SP 02, SP 03

Počet vzoriek/sád:

Dátum skúšky:

Overenie použiteľnosti vzoriek – dovolené tolerancie:

**Rozmery, ktoré boli vo forme: d_1 a l**	presnosť merania:<br>2 merania (na koncoch vzorky)<br>dovolená odchýlka: ±1,0 %
**Odchýlka hornej a spodnej plochy: d_2**	presnosť merania:<br>3 merania: (na koncoch a v strede vzorky)<br>dovolená odchýlka: ± 0,5 %
**Rovinnosť zaťažovanej plochy**	dovolená odchýlka: ± 0,0006d
**Kolmosť bočných stien hranolu k dolnej základni tak, ako bola vyrábaná**	dovolená odchýlka: ± 0,5 mm
**Priamosť zaťažovanej stykovej plochy**	dovolená odchýlka: ± 0,2 mm
**Opracovanie vzorky**	Rezanie, zabrúsenie

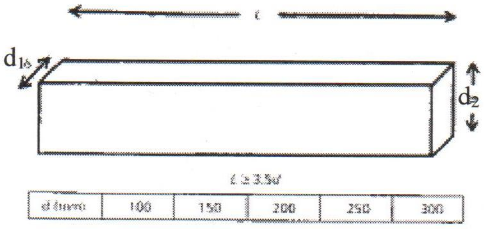

Tabuľka nameraných hodnôt:

Označ. vzorky	Rozmery d_1 x d_2 x l (mm)			Rovinn. plochy [mm]	Kolmosť steny [mm]	Priamosť zaťažovanej plochy [mm]	Hmotnosť (kg)	Max. zaťaženie (N)	Pevnosť (MPa)
B-1-B	100,3	99,8	400,1				8,969	18,089	5,586
	100,2	98,2	400						
	100,1	97,4	400						
Priemer	100,2	98,5	400						
Splnenie kritérií									
B-2-B	102,5	100,2	399,5				9,283	17,552	5,142
	102,3	100,2	399,8						
	101,9	100,2	399						
Priemer	102	100,2	399,4						

Splnenie kritérií									
B-3-B	99,9	102,7	400				8,885	17,591	5,293
	99,9	100,9	399,6						
	100,0	98,8	399,0						
Priemer	99,9	99,9	399,5						
Splnenie kritérií									

Poznámky:

Skúšku vykonal:

Kontroloval:

B-1 97,9 96,0 400 — 8,939 — 306,291 30,629
97,2 96,6 400
97,2 96,9 399,8
97,4 96,5 399,9

B-2 97,1 100,4 399,5 — 9,04 — 216,297, 22,048
97,9 101,7 399,5
97,0 100,3 400
97,3 100,8 399,7

B-3 97,0 94,8 399,5 — 8,825 — 331,062, 33,755
97,7 93,5 399,5
97,7 93,2 400
97,5 93,8 399,7

B-9 100,2 99,2 400 — 9,064 — 323,96 32,691
100 98,5 400,1
100,1 99,3 400,1
100,1 99 400,1

320,421
30% → 96,1263

B-1-E	97,0	94,8	399,5		
	97,1	94,6	400,0	8,975	cca 32 MPa
	97,1	94,2	399,8		
	97,1	94,5	399,8		
B-2-E	97,0	98,0	399,5		
	97,0	97,4	400,0	9,127	cca 29.3 MPa
	97,6	98,6	400,0		
	97,2	98	399,8		
B-3-E	97,7	96,4	400,1		
	97,8	98,2	399,8	9,11	cca 30 MPa
	97,8	97,4	399,7		
	97,8	97,3	399,9		
B-4-E	97,7	100,1	400,15		
	97,8	100,2	400,05	9,042	cca 27 MPa
	98,2	100,9	400,05		
	97,9	100,4	400,1		

05.10.2021
