# Supplementary material for: An experimental and numerical study of twin dowel type shear connector
Source: Sci Rep. 2023 Feb 21;13:3071. doi: 10.1038/s41598-023-30005-3 (PMC9945454; doi:10.1038/s41598-023-30005-3)
Supplement: Supplementary file 1 — Supplementary Information. [file 41598_2023_30005_MOESM1_ESM.zip › Raw_data/Cubes.pdf]

4. 10. 2021
beton+F → 4. 6. 2021

SPRIEVODNÝ LIST ZÁKAZKY
č.:

Druh skúšky: Pevnosť v tlaku
Pevnosť v priečnom ťahu vzoriek tvaru kocka

Skúšobný postup č.: SP 01, SP 03

Počet vzoriek/sád:

Dátum skúšky:

Overenie použiteľnosti vzoriek – dovolené tolerancie:

**Rozmery, ktoré boli vo forme: d_1 a d_2**	presnosť merania:<br>2 merania (na koncoch vzorky)<br>dovolená odchýlka: ± 1,0 %
**Odchýlka hornej a spodnej plochy: d_3**	presnosť merania:<br>2 merania: (na koncoch vzorky)<br>dovolená odchýlka: ± 1,5 %
**Rovinnosť zaťažovanej plochy**	dovolená odchýlka: ± 0,0006d
**Kolmosť bočných stien kocky k dolnej základni tak, ako bola vyrábaná**	dovolená odchýlka: ± 0,5 mm
**Opracovanie vzorky**	Rezanie, zabrúsenie

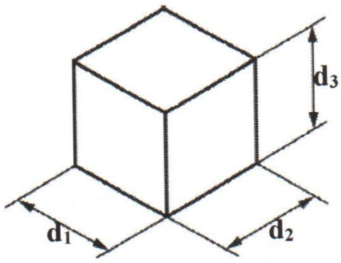

d, mm	100	150	200	250	300

Tabuľka nameraných hodnôt:

Označ. vzorky	Rozmery $d_1 \times d_2 \times d_3$ (mm)			Rovinn. plochy [mm]	Kolmosť steny [mm]	Hmotnosť (kg)	Max. zaťaženie (N)	Pevnosť (MPa)
G-1	150,0	153,2	151,9			7,797	996,89	43,579
	150,2	152,4	151,8					
	150,4	151,2	152,7					
Priemer	150,2	152,3	152,1					
Splnenie kritérií			-			-	-	-
G2	149,9	153,1	150,1			7,766	1091	47,688
	150,3	152,6	150,2					
	150,2	151,9	150,0					

150,1 152,5 150,1

C-2-T	151,4	150,1	150,0
	151,5	150,0	150,1
	151,5	150,2	149,9
	151,5	150,1	150,0

7,701

~~[illegible]~~
118,083

5,145

Priemer								
Splnenie kritérií			-			-	-	-
C-3	150,8	150,0	150,1			7,732	1124,45	49,382
	151,0	150,5	150,0					
	152,8	150,9	150,6					
Priemer	151,3	150,5	150,2					
Splnenie kritérií			-			-	-	-

Poznámky:

Skúšku vykonal: Ing. P. Orolin, PhD.

Kontroloval:

C-4 150,2 150,3 151,2
150,3 149,6 151,0
150,3 149,2 150,2
150,3 149,7 150,8
7,679 1145,68 50919

149,9 150,2 150,1
C-1-T 150,9 150,4 150,1
152,3 150,4 150,2
151 150,3 150,1
7,654 113,325 5,037

C-3-T 149,9 150,0 154,2
149,9 149,9 154,8
149,9 149,9 155,0
149,9 149,9 154,6
7,823 134,234 6,108
