# Supplementary material for: An experimental and numerical study of twin dowel type shear connector
Source: Sci Rep. 2023 Feb 21;13:3071. doi: 10.1038/s41598-023-30005-3 (PMC9945454; doi:10.1038/s41598-023-30005-3)
Supplement: Supplementary file 1 — Supplementary Information. [file 41598_2023_30005_MOESM1_ESM.zip › Raw_data/Cylinders.pdf]

SPRIEVODNÝ LIST ZÁKAZKY
č.:

Druh skúšky: Pevnosť v tlaku vzoriek tvaru valec
Pevnosť v priečnom ťahu vzoriek tvaru valec
Skúšobný postup č.: SP 01, SP 03
Použité meradlá: Posuvné meradlo 250 mm. (inr. 1337/07)
Nožové pravítko (inr. 249/03)
Uholník príložný (inr. N 413/01)
Sparomer (inr. N 414/01)

Počet vzoriek/sád: **Dátum skúšky:**

Overenie použiteľnosti vzoriek – dovolené tolerancie:

**Priemer d**	presnosť merania: 0,05 mm<br>dvojitým vzájomne kolmým meraním na koncoch vzorky<br>dovolená odchýlka: ±1,0 %
**Dĺžka l**	presnosť merania: 0,5 mm<br>2 merania: (maximálna a minimálna dĺžka)<br>dovolená odchýlka: ± 5,0 %
**Rovinnosť zaťažovanej plochy**	dovolená odchýlka: ± 0,0006d
**Kolmosť steny valca vzhľadom na koncové plochy**	dovolená odchýlka: ± 0,007d
**Priamosť povrchovej priamky valca (len pri priečnom ťahu)**	dovolená odchýlka: ± 0,2 mm
**Opracovanie vzorky**	Rezanie, zabrúsenie, koncovanie zaťaž. plôch

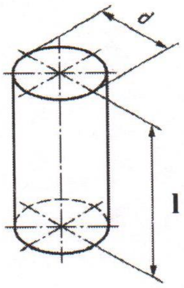

d, mm	100	113 [a]	150	200	250	300
[a] Tejto hodnote zodpovedá zaťažovaná plocha 10 000 mm².						

Tabuľka nameraných hodnôt:

Označ. vzorky	Priemer d [mm]		Dĺžka l [mm]		Hmotn. [kg]	Rovinn. plochy [mm]	Kolmosť steny [mm]	Priamosť valcovej plochy	Poloha výstuže [mm]		Max. zaťaženie [N]	Pevnosť [MPa]
	1. štvrtina	2. štvrtina	Pred úpravou max./ min.	Po úprave max./ min.					Pred úpravou	Po úprave		
CY-1	149,4	149,9	299,5		11,849						141<br>600,[illegible]	34,234
	149,1	149,1	299									

149,3 149,5

Priemer												
Splnenie kritérií									-	-	-	-
C4-2	149,3	149,3	299,5		11,866						641,575	34,645
	149,3	149,3	300									
Priemer	149,3	149,3	299,8									
Splnenie kritérií									-	-	-	-
C4-3	149,4	149,5	299,9		11,93						615,972	35,090
	149,5	149,4	300									
Priemer	149,5	149,5	300									
Splnenie kritérií									-	-	-	-

Poznámky:

Skúšku vykonal:

Kontroloval:
