# Supplementary material for: An experimental and numerical study of twin dowel type shear connector
Source: Sci Rep. 2023 Feb 21;13:3071. doi: 10.1038/s41598-023-30005-3 (PMC9945454; doi:10.1038/s41598-023-30005-3)
Supplement: Supplementary file 1 — Supplementary Information. [file 41598_2023_30005_MOESM1_ESM.zip › Raw_data/drawing.pdf]

Analýza šmykovej odolnosti spriahovacích pásov s modifikovaným kontinuálnym spriahnutím

M 1:10

VZORKA S OCEĽOVOU VÝSTUŽOU, ks: 4

REZ A-A

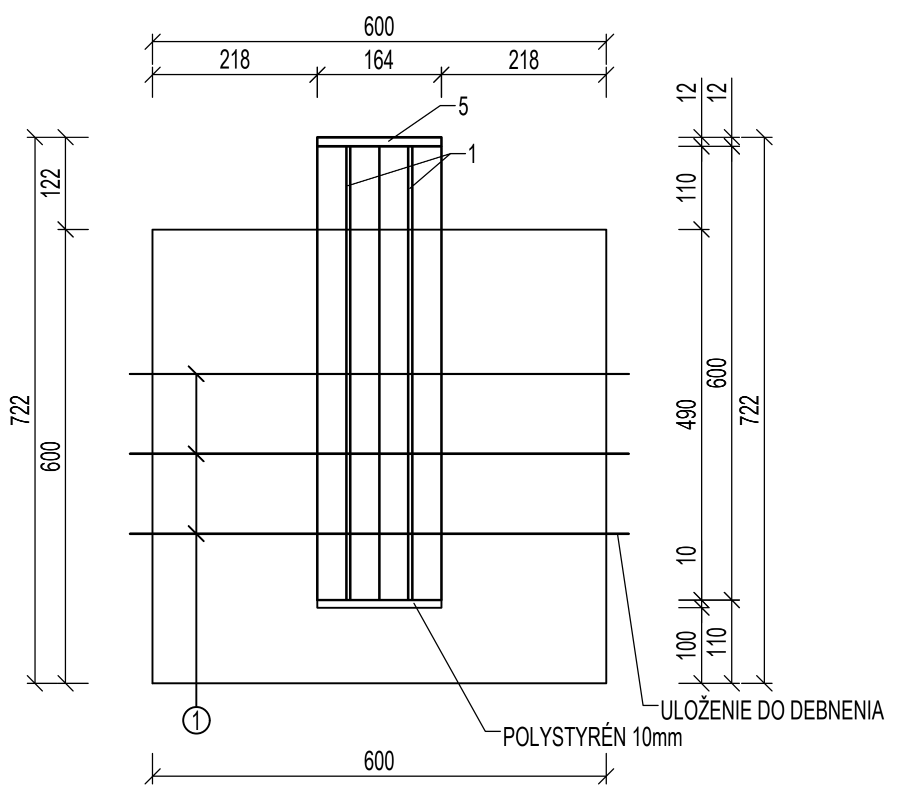

VZORKA S VÝSTUŽOU NA BÁZE SKLA, ks: 4

REZ C-C

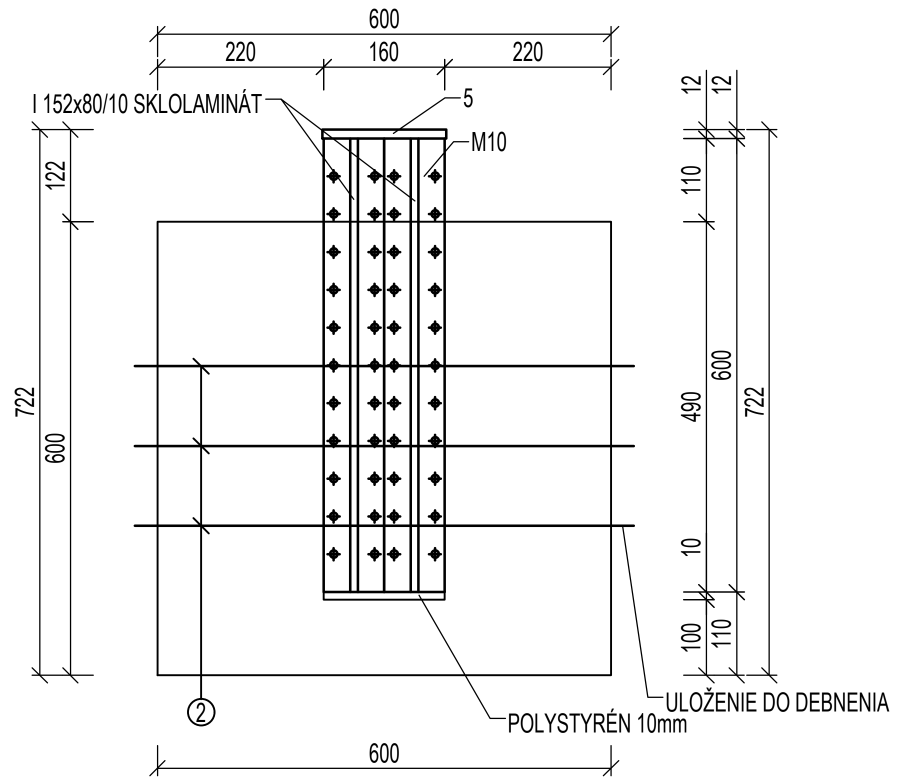

ORIENTAČNÝ VÝKAZ MATERIÁLU

	Počet	Druh	Rozmer	Šírka	Dĺžka	Priemer	Dĺžka	VÁHA		PLOCHA		
pol.	kusov	prof.	profilu	plechu	prvku	trubky	celkom	[kg/m]	Celkom	$[m^2/m]$	Celkom	Akosť
	celkom			B [mm]	L[mm/ks]	D [mm]	[m]		[kg]		$[m^2]$	mat.
1	8	IPE	160		600		4.8	15.80	75.84	0.62	3.0	11373
2	8	Plech	12	100	600		4.8	9.42	45.22	0.12	0.6	11375
3	8	Plech	12	80	600		4.8	7.54	36.17	0.10	0.5	11375
4	8	Plech	10	160	600		4.8	12.56	60.29	0.19	0.9	11375
5	8	Plech	12	164	120		0.96	15.45	14.83	0.04	0.0	11375

Hmotnosť [kg]			232.35
Globálna prirážka [kg]	10%	(skrutky, zvary, odrezky, kotvy)	23.23
Celková hmotnosť [kg]			255.58
Celková náterová plocha $[m^2]$			4.97

SPÔSOB VYREZANIA PÁSU Z IPE 160, I 152x80/10

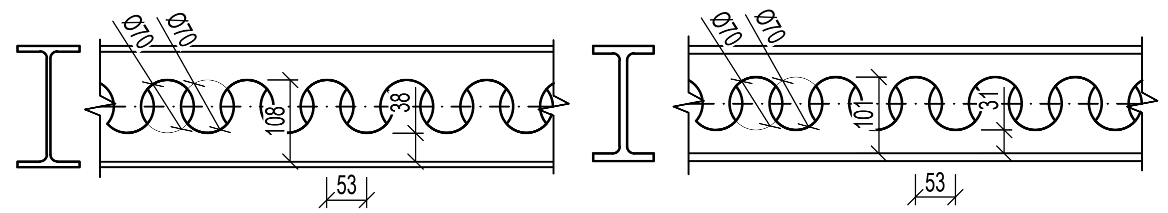

PÔDORYS

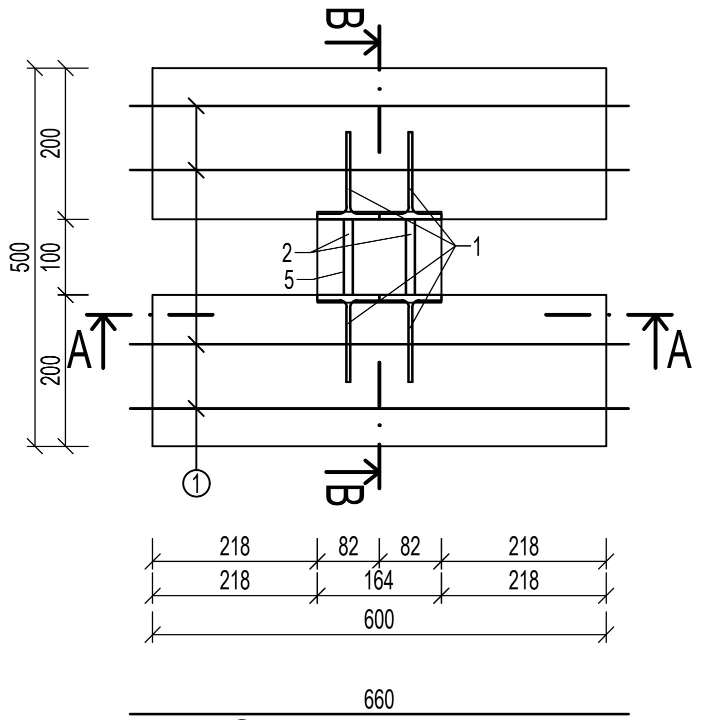

① Ø12R a 100mm, 660mm, ks: 48

REZ B-B

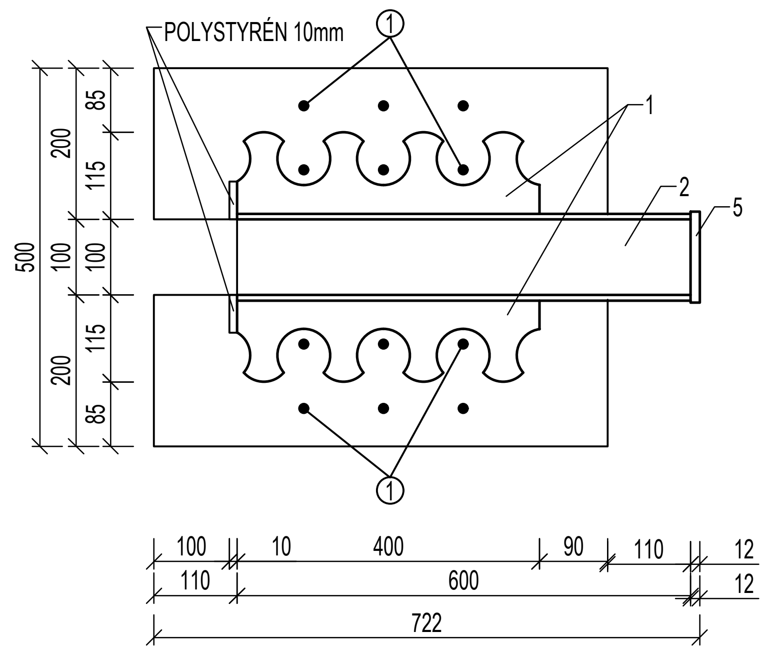

PÔDORYS

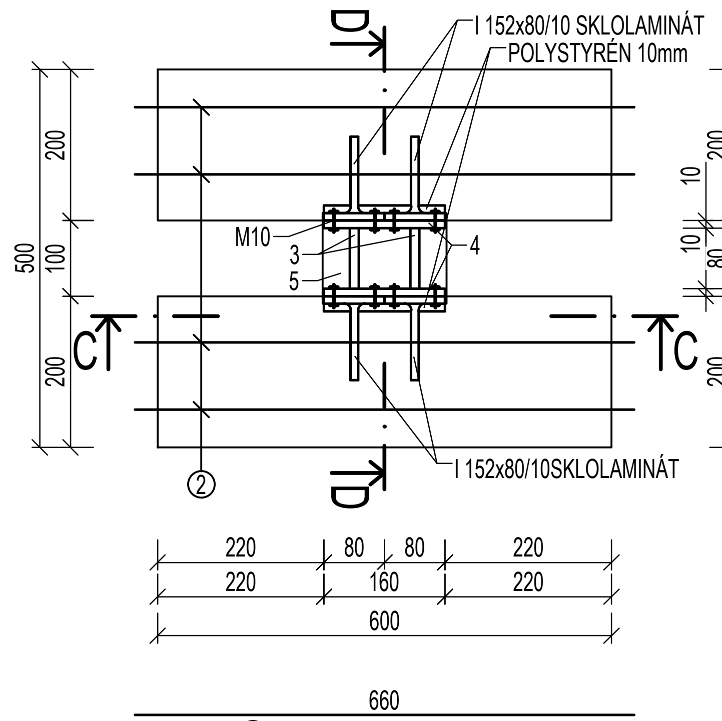

② Ø12 a 100mm, 660mm, ks: 48
GFRP VÝSTUŽ

REZ D-D

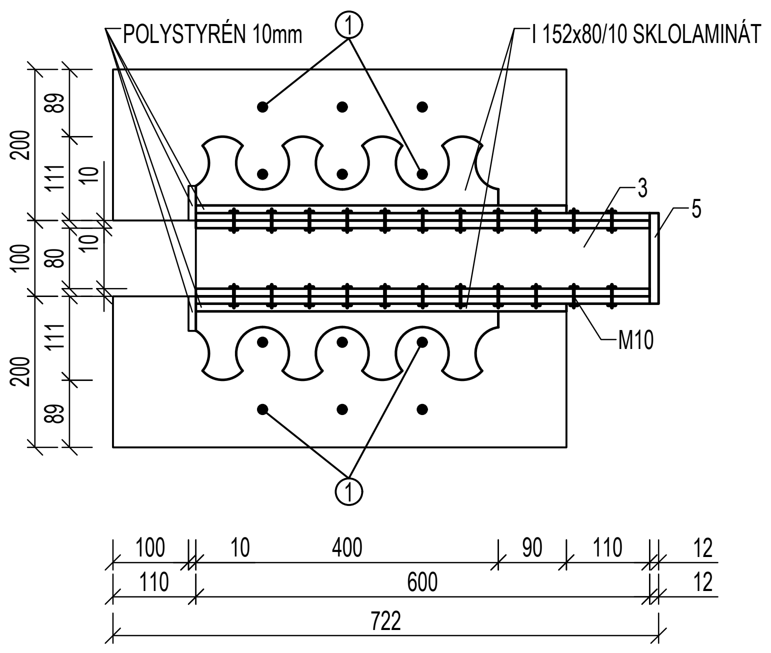

ORIENTAČNÝ VÝKAZ MATERIÁLU SKLOLAMINÁT:
I 152x80/10 dl. 600mm, 8ks

ORIENTAČNÝ VÝKAZ GFRP VÝSTUŽE:
p.č. 2, R12, dl. 660mm, 48ks

ORIENTAČNÝ VÝKAZ SKRUTIEK:
M10 352ks

MATERIÁL:
- OCEĽ TRIEDY S355
- BETÓN TRIEDY C30/37
- BETONÁRSKA VÝSTUŽ B500
- SKLOLAMINÁT
- GFRP VÝSTUŽ

Výkaz betonárskej výstuže

Prvok			
Označenie	dĺžka [mm]	počet [ks]	R12 [mm]
1	660	48	31680
spolu [m]			31.68
jednotková hmotnosť [kg]			0.8878
hmotnosť [kg]			28.125504
celkom hmotnosť [kg]			28.125504

ZODPOVEDNÝ RIEŠTEĽ Ing. Patrícia Vaňová	RIEŠITELIA Ing. Patrícia Vaňová, Ing. Jakub Bartuš, Ing. Łukasz Skrętkowicz	TECHNICKÁ UNIVERZITA V KOŠICIACH STAVEBNÁ FAKULTA
NAVRHOVALA Ing. Patrícia Vaňová		
VYPRACOVALA Ing. Patrícia Vaňová		
**Analýza šmykovej odolnosti spriahovacích pásov s modifikovaným kontinuálnym spriahnutím**	DÁTUM 01/2021	FORMÁT 3xA4
Pretláčacie vzorky	Č. VÝKRESU ST-01	MIERKA 1:10
