# Supplementary material for: An experimental and numerical study of twin dowel type shear connector
Source: Sci Rep. 2023 Feb 21;13:3071. doi: 10.1038/s41598-023-30005-3 (PMC9945454; doi:10.1038/s41598-023-30005-3)
Supplement: Supplementary file 1 — Supplementary Information. [file 41598_2023_30005_MOESM1_ESM.zip › Raw_data/framework.pdf]

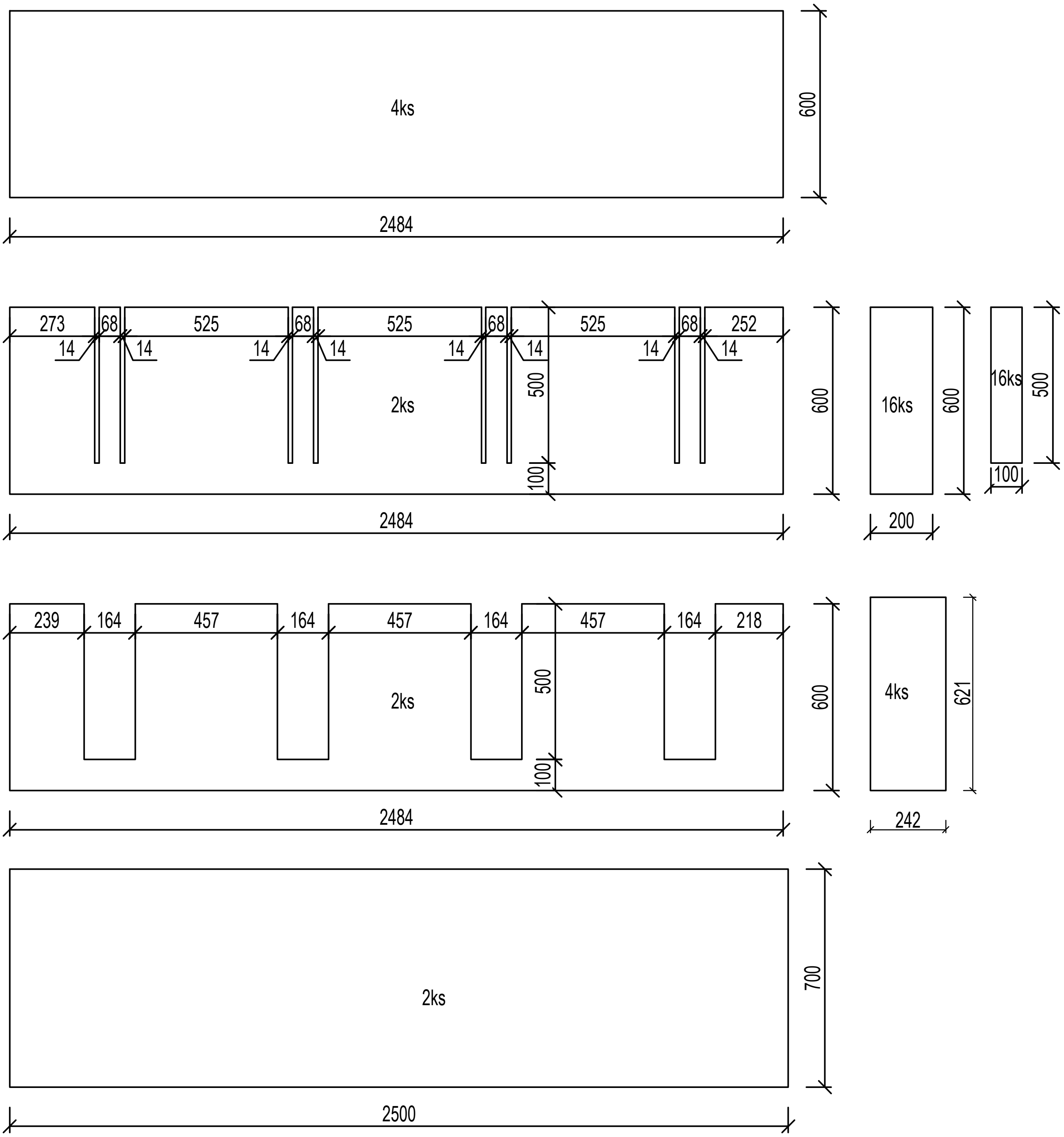

LDTD hr. 21mm

ZODPOVEDNÝ RIEŠTEĽ<br>Ing. Patrícia Vaňová	RIEŠITELIA<br>Ing. Patrícia Vaňová<br>Ing. Jakub Bartuš<br>Ing. Łukasz Skrętkowicz	TECHNICKÁ UNIVERZITA<br>V KOŠICIACH<br>STAVEBNÁ FAKULTA
NAVRHOVALA<br>Ing. Patrícia Vaňová		
VYPRACOVALA<br>Ing. Patrícia Vaňová		

Analýza šmykovej odolnosti spriahovacích pásov s modifikovaným kontinuálnym spriahnutím

DÁTUM	FORMÁT
02/2021	1xA4

Výkres materiálu debnenia

Č. VÝKRESU	MIERKA
ST-02	1:20
